# Progressive waves of IL-1β release by primary human monocytes via sequential activation of vesicular and gasdermin D-mediated secretory pathways

**DOI:** 10.1038/s41419-018-1121-9

**Published:** 2018-10-23

**Authors:** Claudia Semino, Sonia Carta, Marco Gattorno, Roberto Sitia, Anna Rubartelli

**Affiliations:** 1 Protein Transport and Secretion Unit, IRCCS Ospedale San Raffaele/Università Vita-Salute San Raffaele, 20132 Milan, Italy; 2 Cell Biology Unit, Ospedale Policlinico San Martino, 16132 Genoa, Italy; 3 Clinica Pediatrica e Reumatologia, “G. Gaslini” Scientific Institute, 16147 Genoa, Italy

## Abstract

IL-1β is an essential cytokine, but its release needs to be strictly controlled to avoid severe inflammatory manifestations. Lacking a signal sequence, IL-1β does not follow the endoplasmic reticulum-Golgi route. Several pathways have been proposed to mediate its release. One involves the translocation of pro-IL-1β into intracellular vesicles of lysosomal origin that eventually fuse with the plasma membrane. Another exploits pores formed on the plasma membrane upon proteolytic cleavage of gasdermin D (GSDMD). Here we investigated how primary monocytes—the main source of IL-1β in humans—control IL-1β release in response to pro-inflammatory stimuli of increasing intensity and found that two different routes are induced depending on the strength of activation. Triggering of Toll-like receptor 4 (TLR4) by LPS induces slow IL-1β release through LAMP2A^+^ vesicles. In contrast, the simultaneous stimulation of TLR2, TLR4 and TLR7/8 drives high levels of ROS, GSDMD cleavage and faster IL-1β secretion. Drugs blocking ROS production prevent GSDMD cleavage supporting a role of oxidative stress in GSDMD-mediated secretion. Singly stimulated monocytes undergo apoptosis, whereas triple stimulation triggers pyroptosis, which might amplify inflammation. In both cases, however, IL-1β secretion precedes cell death. Inhibition of caspases 4/5 prevents GSDMD cleavage and pore-mediated secretion, but not vesicular release. The two pathways also display other distinct pharmacologic sensitivities that reflect the underlying mechanisms. Remarkably, single TLR4 stimulation is sufficient to activate massive, GSDMD-mediated IL-1β secretion in monocytes from patients affected by Cryopyrin Associated Periodic Syndrome (CAPS), an autoinflammatory disease linked to *NLRP3* mutations. The exaggerated sensitivity to activation correlates with high basal ROS levels in CAPS monocytes. In conclusion, the vesicular pathway limits IL-1β release upon low pathogen load while stronger stimulation or concomitant cell stress induce instead uncontrolled secretion via GSDMD leading to detrimental inflammatory manifestations.

## Introduction

IL-1β is essential for recovery from infections and trauma^1^. However, its dysregulated production is implicated in many acute and chronic diseases, acquired^2^ and inherited^3^. The cytokine is synthesized upon Toll-like receptor (TLR) triggering as a cytosolic precursor (pro-IL-1β), which is processed by caspase 1 upon activation of the inflammasome, an intracellular multimeric protein complex that assembles upon stimulation by various signals^4^.

IL-1β lacks a secretory signal sequence, a feature shared by other ‘leaderless’ secretory proteins^5^. After the early observation that IL-1β is actively secreted by human monocytes through a route alternative to the ER-Golgi pathway^6^, several secretory pathways were proposed^7^ involving secretory lysosomes^8,9^ exosomes^10^, microvesicles^11,12^, and autophagic vesicles^13–17^, possibly through a mechanism similar to chaperone-mediated autophagy (CMA)^17,18^.

More recently, another route for IL-1β release was disclosed in mouse macrophages and myelomonocytic cell lines, involving gasdermin-D (GSDMD) and pyroptosis^19,20^. Infection with intracellular pathogens, mimicked by LPS transfection, activates the non-canonical inflammasome, namely, murine caspase 11 or its human homologs caspase 4/5^21,22^. In turn, these caspases cleave GSDMD, generating toxin-like peptides that form pores on the plasma membrane mediating the selective secretion of mature IL-1β^20,23^. When cells are exposed to the strong NLRP3 inflammasome activator nigericin^19,24^, also caspase 1 can cleave GSDMD with a mechanism independent of its role as IL-1β converting enzyme^19^.

Thus, how is IL-1β released is still debated, even due to the many differences existing in IL-1β secretion by different cell types^4,25–29^. For instance, primary human monocytes do not require a second signal after TLR triggering for processing and secretion of IL-1β, whereas murine macrophages and myelomonocytic cell lines do^30^. Also the way and intensity of stimulation can influence the rate, amount and mechanism(s) of IL-1β secretion. In primary human monocytes, triggering a single TLR induces a self-limiting redox response resulting in slow release of IL-1β^31,32^. Instead, co-stimulation of three different TLRs causes early secretion of abundant IL-1β sustained by a strong ROS production, that degenerates in oxidative stress^32^. Intense and fast IL-1β secretion, responsible for devastating inflammatory manifestations, and oxidative stress characterize also monocytes from patients affected by Cryopyrin Associated Periodic Syndrome (CAPS)^33,34^, an inherited autoinflammatory disorder linked to mutations in NLRP3^3^.

On these bases, we surmised that human monocytes can use more than one route during inflammatory responses, integrating different cues (i.e., type of stimulus, metabolic state and/or basal stress). Accordingly, our results reveal that in healthy monocytes mild stimuli (i.e., single TLR triggering) induce accumulation of pro-IL-1β in the cytosol and in LAMP2A^+^ secretory lysosomes, with slow release of IL-1β before apoptotic death. Multiple TLR engagement triggers instead GSDMD cleavage, with faster and abundant IL-1β release followed by pyroptosis. Remarkably, in CAPS monocytes, already stressed at baseline^33,34^, LPS alone is sufficient to induce GSDMD cleavage. Thus, the intensity of proinflammatory stimuli in healthy monocytes, or the presence of stress in monocytes carrying mutant NLRP3, induce a shift from a vesicular pre-apoptotic pathway to a GSDMD-dependent secretory route that culminates in pyroptosis.

## Results

### Hyperstimulation of primary monocytes induces GSDMD cleavage

**Fig. 1:**
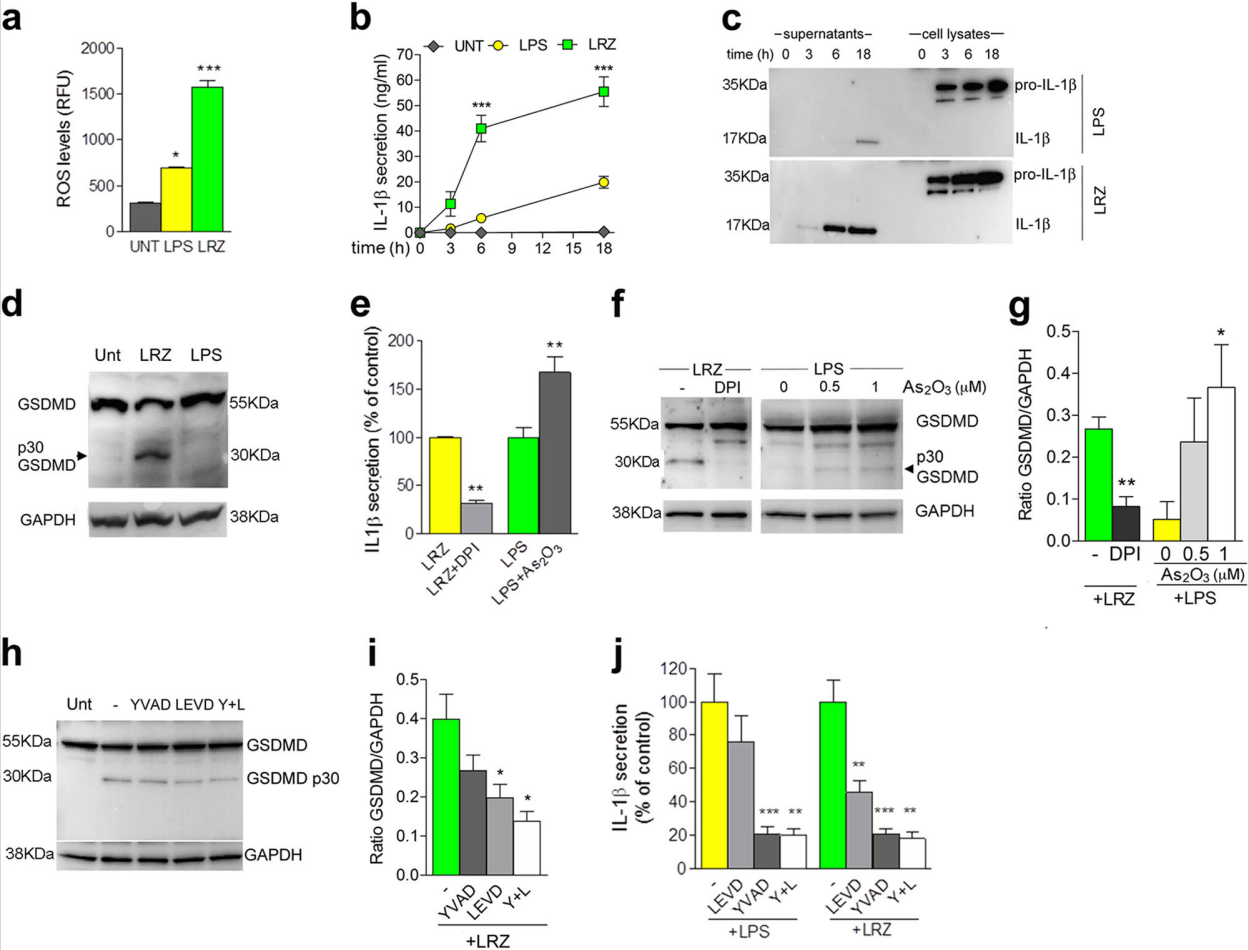
Costimulation of TLR2, TLR4, and TLR7/8 induces ROS production, GSDMD cleavage and IL-1β hypersecretion. **a** Intracellular ROS quantified by H_2_DCF-DA staining in monocytes unstimulated or stimulated for 1 h as indicated. *N* = 3. Data are expressed as RFU. **b**, **c** Monocytes from healthy donors were incubated with LPS alone or with LPS, R848, Zymosan (LRZ) for the indicated times (hours). (**b**) Time course of IL-1β secretion, quantified by ELISA. *N* = 6. Data are expressed as ng/ml (mean ± SEM). (**c**) One representative Western blot analysis (out of 3 performed) of intracellular and extracellular IL-1β at various times of exposure to LPS or LRZ. **d** One representative Western blot (out of 5 performed) showing GSDMD cleavage in cell lysates from monocytes cultured 6 h untreated (Unt) or with LRZ or LPS. Arrowhead indicates the p30 N-terminal domain of GSDMD. GAPDH served as loading control. **e** Secretion of IL-1β by monocytes stimulated with LRZ or LPS in the presence or absence of DPI (20 μM) or As_2_O_3_ (1 μM) as indicated. Data are expressed as the percent of IL-1β secreted by LPS or LRZ stimulated monocytes exposed to DPI or As_2_O_3_ relative to secretion without drugs. **f, g** Western blot analyses of GSDMD cleavage in monocytes stimulated 6 h with LRZ or LPS in the presence or absence of DPI (20 μM) or As_2_O_3_ as indicated. **f** One representative western blot out of 3 performed; arrowheads indicate p30 GSDMD. **g** Ratio of GSDMD p30/GAPDH obtained by quantitative densitometry of the 3 western blots like the one shown in **f** (mean ± SEM). **h, i** Western blot analyses of GSDMD cleavage in monocytes stimulated 6 h with LRZ or LPS in the presence or absence of Ac-YVAD, Z-LEVD or Ac-YVAD plus Z-LEVD (Y + L). **h** One representative western blot out of 3 performed; **i** Ratio of GSDMD p30 /GAPDH obtained by quantitative densitometry of the 3 western blots like the one shown in **h** (mean ± SEM). **j** Caspase dependency of IL-1β secretion. Monocytes were stimulated with LPS or LRZ in the presence or absence of Ac-YVAD, Z-LEVD or Ac-YVAD plus Z-LEVD (Y + L) and analyzed as above. Data are expressed as in **e**. Data information: One-way ANOVA (**a, b, h, i**) or Unpaired *t*-test (**e, g**) were used for significance (*P < 0.05, **P < 0.01, ***P < 0.001)

In primary human monocytes, co-stimulation of TLR4, TLR7/8, and TLR2 with the cognate agonists (LPS + R848 + Zymosan, [LRZ]) induces stronger ROS production (Fig. 1a) and higher secretion of IL-1β than LPS alone (32, Fig. 1b, c).

Remarkably, while only the uncleaved form of GSDMD is detected in unstimulated and singly stimulated monocytes (Fig. 1d), triply stimulated monocytes exhibit also the cleaved p30 GSDMD (Fig. 1d), corresponding to the pore-forming N-terminal domain^19,20^. In LRZ-stimulated monocytes, the flavoprotein inhibitor DPI prevented not only the increase of intracellular ROS and secreted IL-1β (31, Fig. 1e) but also the generation of p30 GSDMD (Fig. 1f, g). Conversely, the pro-oxidant drug As_2_O_3_ triggered the appearance of the p30 GSDMD band (Fig. 1f, g) and increased IL-1β secretion (Fig. 1e) in LPS-stimulated monocytes, confirming the role of redox stress in GSDMD cleavage.

Caspases 4/5 and 1 have been proposed as executors of GSDMD cleavage^20–24^. In agreement, the caspase 4/5 inhibitor Z-LEVD prevented by about 50% the generation of p30 GSDMD in LRZ-stimulated monocytes (Fig. 1h, i). The caspase 1 inhibitor Ac-YVAD induced only a slight decrease of GSDMD cleavage (Fig. 1h, i) but the combined exposure to both inhibitors produced an additive inhibitory effect (Fig. 1h, i). Consistently, IL-1β secretion by LRZ-treated monocytes was halved by caspase 4/5 inhibitors that, on the contrary, did not affect IL-1β secretion by LPS-treated monocytes (Fig. 1j). In keeping with the primary function of caspase 1 as IL-1β converting enzyme^7^, a drastic reduction of mature IL-1β secretion was observed in both singly and triply stimulated monocytes exposed to the caspase 1 inhibitor Ac-YVAD (Fig. 1j). Neither Z-LEVD nor Ac-YVAD impaired pro-IL-1β accumulation (Fig. S1).

**Fig. 2:**
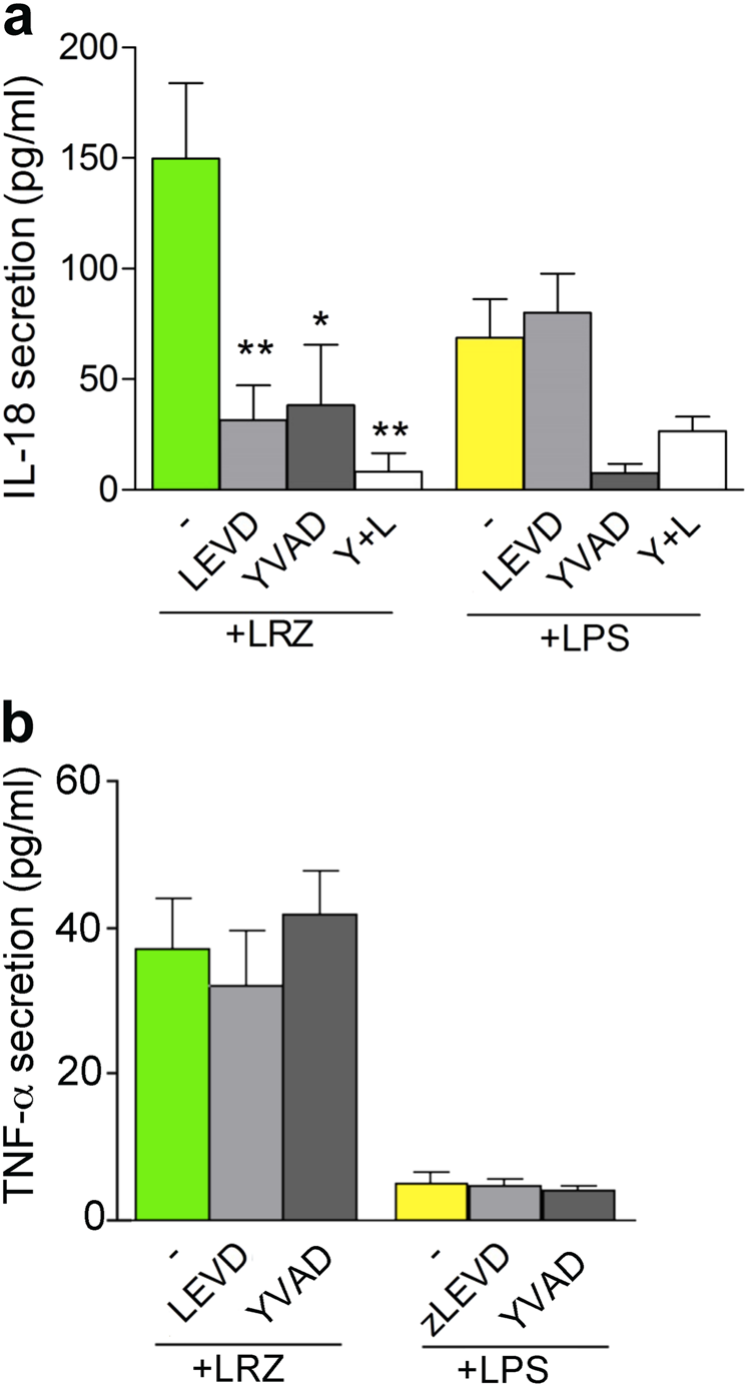
Caspase dependency of LRZ-induced IL-18 secretion **a, b** IL-18 (**a**) and TNF-α (**b**) secreted in supernatants of monocytes stimulated 18 h with LRZ or LPS in the presence or absence of Ac-YVAD, Z-LEVD, or both (Y + L), as indicated (*N* = 3). Data are expressed as pg/ml (**a**) or ng/ml (**b**), mean ± SEM. Data information: One-way ANOVA was used for significance (*P < 0.05, **P < 0.01)

Notably, also the secretion of IL-18, that like IL-1β is leaderless and is processed by caspase-1, was enhanced by triple stimulation and inhibited by Z-LEVD, Ac-YVAD and, at higher extent, by the combination of the two (Fig. 2a). In contrast, the secretion of a leader sequence bearing cytokine such as TNF-α^32^, was unaffected by the caspase inhibitors (Fig. 2b).

### Hyperstimulation of primary monocytes culminates with pyroptosis

**Fig. 3:**
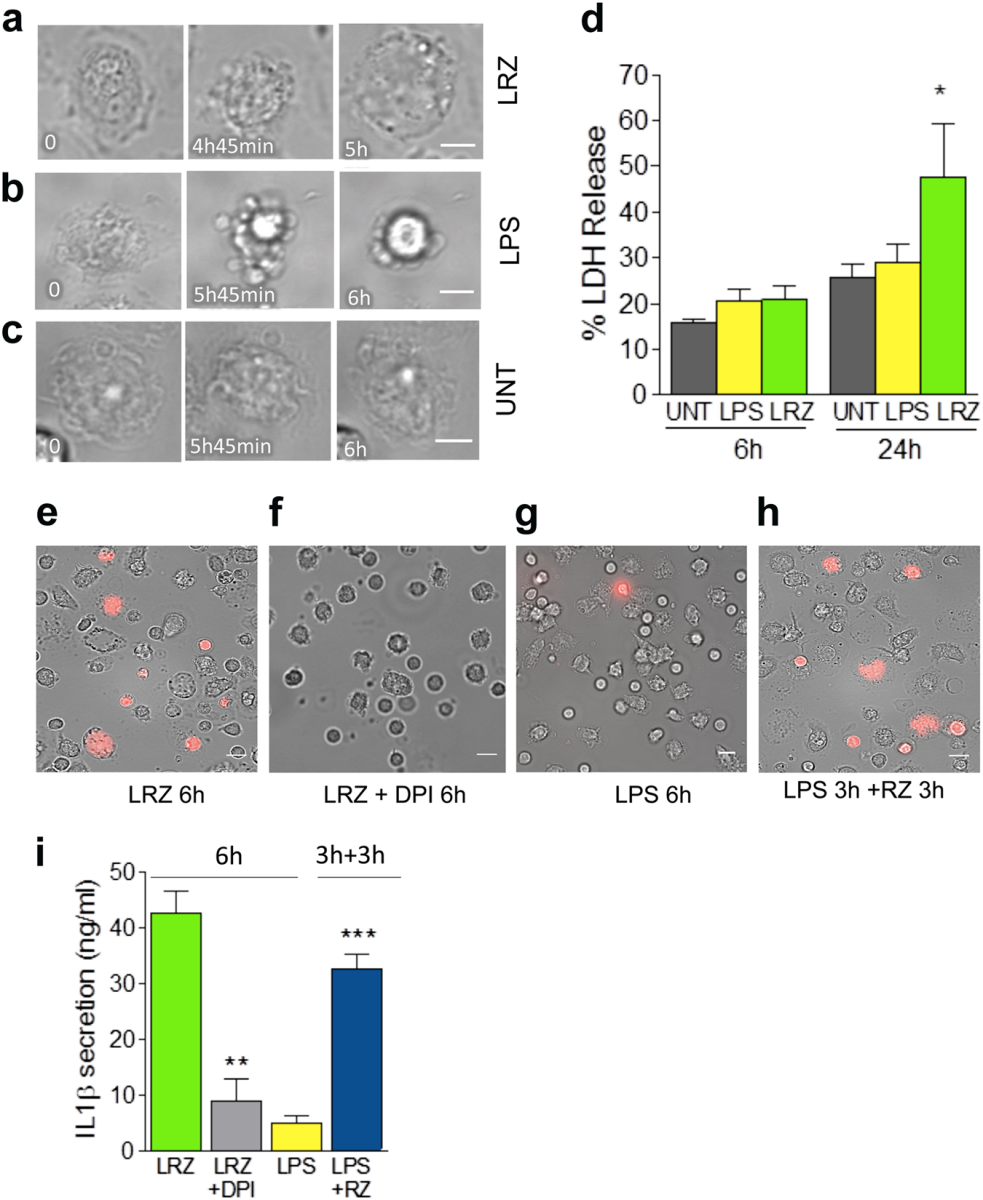
Multiple stimulation induces pyroptosis whereas single stimulation induces apoptosis **a–c** Monocytes were exposed to medium alone or supplemented with LPS or LRZ and analyzed by live cell microscopy for 6 h (*N* = 3). Images were taken every 3 min. Scale bar, 5 μm. **a–b** Morphologic changes of representative LRZ-stimulated (**a**) LPS-stimulated (**b**) or unstimulated (UNT, **c**) monocytes. Little if any morphologic changes are seen in unstimulated monocytes within this time frame. Timestamps are indicated in each image. **d** Quantification of LDH in supernatants from untreated (Unt), LPS or LRZ-stimulated monocytes. *N* = 6. Data are expressed as percent of released LDH vs. total LDH, mean ± SEM. **e-h** Live cell microscopy images of propidium iodide (PI) staining of monocytes exposed 6 h to LRZ in the absence (**e**) or presence of DPI (**f**), or to LPS (**g**), or for 3 h to LPS alone followed by 3 additional h with also R848 and zymosan (RZ) (**h**). (*N* = 3). **i** IL-1β secreted by cells stimulated 6 h with LRZ, in the presence or absence of DPI, or 6 h with LPS, or 3 h with LPS followed by 3 h with R848 and zymosan. Data are expressed as ng/ml (mean ± SEM). *N* = 3. Data information: In (**d**) One-way ANOVA and in (**i**) Unpaired *t*-test was used for significance (*P < 0.05, **P < 0.01, ***P < 0.001)

Monocytes stimulated with LRZ (Fig. 3a and Movie S1) undergo swelling and blebbing culminating with cytoplasmic flattening and burst, a pattern consistent with pyroptosis^35,36^. In contrast, most LPS-stimulated monocytes do not display pyroptotic cell death, but undergo apoptosis (Fig. 3b and Movie S2). The morphological changes in LRZ-stimulated monocytes started slightly earlier than those observed in LPS-stimulated cells. No significant morphologic changes were detected in untreated monocytes within the same time frames (Fig. 3c and Movie S3). After 18 h of incubation, dead cell number was highly variable in the different donors but consistently more numerous in triply-stimulated (>60%) than singly-stimulated monocytes (about 45%).

Extracellular lactate dehydrogenase (LDH), a marker of aspecific membrane leakage, was equally low in all culture conditions (Fig. 3d), although IL-1β secretion was already almost at plateau in hyperstimulated cells (see Fig. 1b). In agreement with several reports (reviewed in 37), LDH release remained low in supernatants of LPS-treated cells for the duration of the experiment, consistent with apoptosis being their fate. In LRZ-treated cell supernatants LDH increase was detected only at later times (24 h, Fig. 3d).

Unlike LDH, the small molecule propidium iodide (PI) can pass through GSDMD pores: coherently, the permeability to PI at 6 h was high in LRZ-treated monocytes (Fig. 3e) but almost absent in LPS stimulated cells (Fig. 3g). The ROS inhibitor DPI that blocks GSDMD cleavage (see Fig. 1f), also prevented pyroptosis, as indicated by the low PI staining (Fig. 3f).

Interestingly, the commitment to slow IL-1β secretion followed by apoptotic cell death triggered by LPS can be overruled by subsequent hyperstimulation. As shown in Fig. 3h and Movie S4, the addition of R848 and zymosan for 3 h after 3 h of LPS alone triggered the pyroptotic pathway with PI staining of a high number of cells and high levels of secreted IL-1β (Fig. 3i). Also cells stimulated with LPS alone for 6 h followed by 3 h with RZ underwent pyroptosis and hypersecretion of IL-1β whereas longer stimulations with LPS alone prevented the switch to pyroptotic secretion (not shown).

### Different intracellular traffic of pro-IL-1β upon single or multiple TLR engagement

The differences in ROS production, GSDMD cleavage, rate of IL-1β secretion and type of cell death indicate that the stimulation with LPS alone or LRZ induce distinct secretory routes. Therefore, we investigated the cellular localization of pro-IL-1β.

**Fig. 4:**
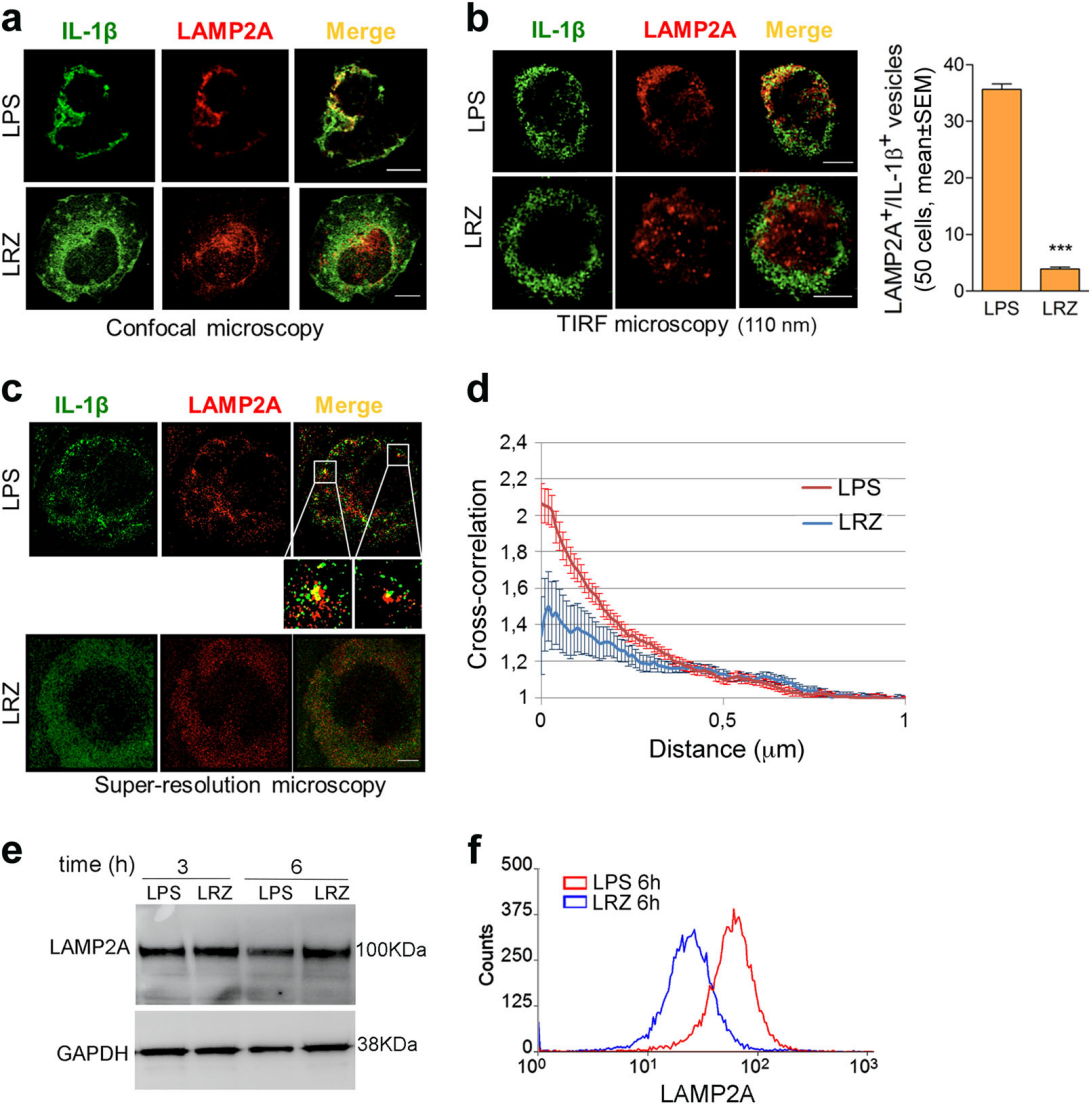
Subcellular localization of IL-1β and LAMP2A in LPS and LRZ-stimulated monocytes. **a** Confocal microscopy of representative monocyte stimulated 6 h with LPS (upper panel) or LRZ (lower panel), stained for IL-1β and LAMP2A as indicated (*N* = 6). Scale bar, 5 μm. One single stack of Z-stack series is shown (Z = 4 for LPS and Z = 5 for LRZ). **b** Left-hand panels: TIRF microscopy of representative monocyte stimulated with LPS (upper panel) or LRZ (lower panel) and stained as in **a** (*N* = 6). Penetration depth = 110 nm. Scale bar, 5 μm. Right-hand panel: quantification of LAMP2A^+^/IL-1β^+^ vesicles found by TIRF analysis, in 50 LPS or LRZ stimulated monocytes, using Laplacian plugins for the extraction of image features (ImageJ) (*N* = 6), mean ± SEM. **c** Direct stochastic optical reconstruction microscopy (dSTORM) of IL-1β and LAMP2A as indicated (*N* = 4). A representative LPS (upper panel) and LRZ-stimulated cell (lower panel) is shown. Scale bar, 2μm. Insets show magnifications to better highlight structures where IL-1β and LAMP2A molecules are close to each other. **d** Cross-correlation (C) between the two channels (green and red, corresponding to IL-1β and LAMP2A fluorescence) as a function of distance. Six cells from 4 independent experiments were analyzed. For each cell, 10 representative ROIs (2 × 2 μm^2^) were chosen in the peripheral region of the cells for the analysis. The closeness of LAMP2A and IL-1β molecules is not random (C > 1) and increased co-clustering at the plasma membrane is higher in LPS-stimulated monocytes (*y* axis, amplitude of the cross correlation). Error bars indicate standard error of C on each ROI in the individual cell. **e** The image shows a representative Western blot (out of 3 performed) comparing the intracellular pools of LAMP2A and GAPDH in monocytes stimulated for 3 or 6 h with LPS or LRZ. **f** Surface expression of LAMP2A increases upon a 6 h stimulation with LPS relative to LRZ (monocytes from a representative subject out of 3 tested are shown). Data information: In **b** (right-hand panel) data are expressed as Mean ± SEM. Unpaired *t*-test was used for significance (***P < 0.001)

Confocal microscopy of LPS-stimulated monocytes revealed that, in addition to a diffuse cytosolic distribution, part of IL-1β co-localized with LAMP2A, a lysosomal membrane protein required for CMA^38^. Double-positive vesicles were easily detectable where cells adhere to the slide (Fig. 4a, upper panel and Movie S5). In LRZ stimulated monocytes, fewer LAMP2A^+^ vesicles were found at the plasma membrane, most of them being scattered throughout the cytosol (Fig. 4a, lower panel and Movie S6) or localized in the perinuclear area (Fig. S2). Co-localization with IL-1β was scarce. These findings were confirmed by TIRF analyses that provided high-contrast images of fluorophores present in the near-membrane region, with a low background from the bulk of the cell, where cytosolic pro-IL-1β is abundant^8^. Clearly, LAMP2A^+^/IL-1β^+^ vesicles concentrated in the proximity of the ventral cell membrane and were much more abundant in LPS-treated than in LRZ-treated monocytes (Fig. 4b). In unstimulated monocytes, no IL-1β is detected while LAMP2A^+^ lysosomes are present (Fig. S3).

Super-resolution microscopy, which reduces the possibility of artefactual co-localization, supported the above data (Fig. 4c, d). Statistical analyses confirmed that LAMP2A/IL-1β co-clustering at the plasma membrane level is higher in LPS-stimulated than in LRZ-stimulated monocytes (Fig. 4d, Paired *t* test *p* < 0.05). These patterns are consistent with the hypothesis that IL-1β accumulates inside LAMP2A^+^ vesicles in LPS (Fig. 4c, upper panel and inset), but not in LRZ-activated monocytes (Fig. 4c, lower panel).

The different co-localization is not due to different levels of intracellular LAMP2A in LPS and LRZ stimulated cells (Fig. 4e). However, surface expression of LAMP2A was higher in single than triple stimulated cells, supporting the externalization of secretory lysosomes (Fig. 4f)

Thus, while in LPS-stimulated monocytes many IL-1β^+^/LAMP2A^+^ lysosomes are present, supporting lysosome-mediated secretion, the small number of double positive structures in hyperstimulated monocytes supports the activation of a different, vesicle-independent secretory pathway.

### Different drug sensitivity of IL-1β secretion by LPS and LRZ-stimulated monocytes

**Fig. 5:**
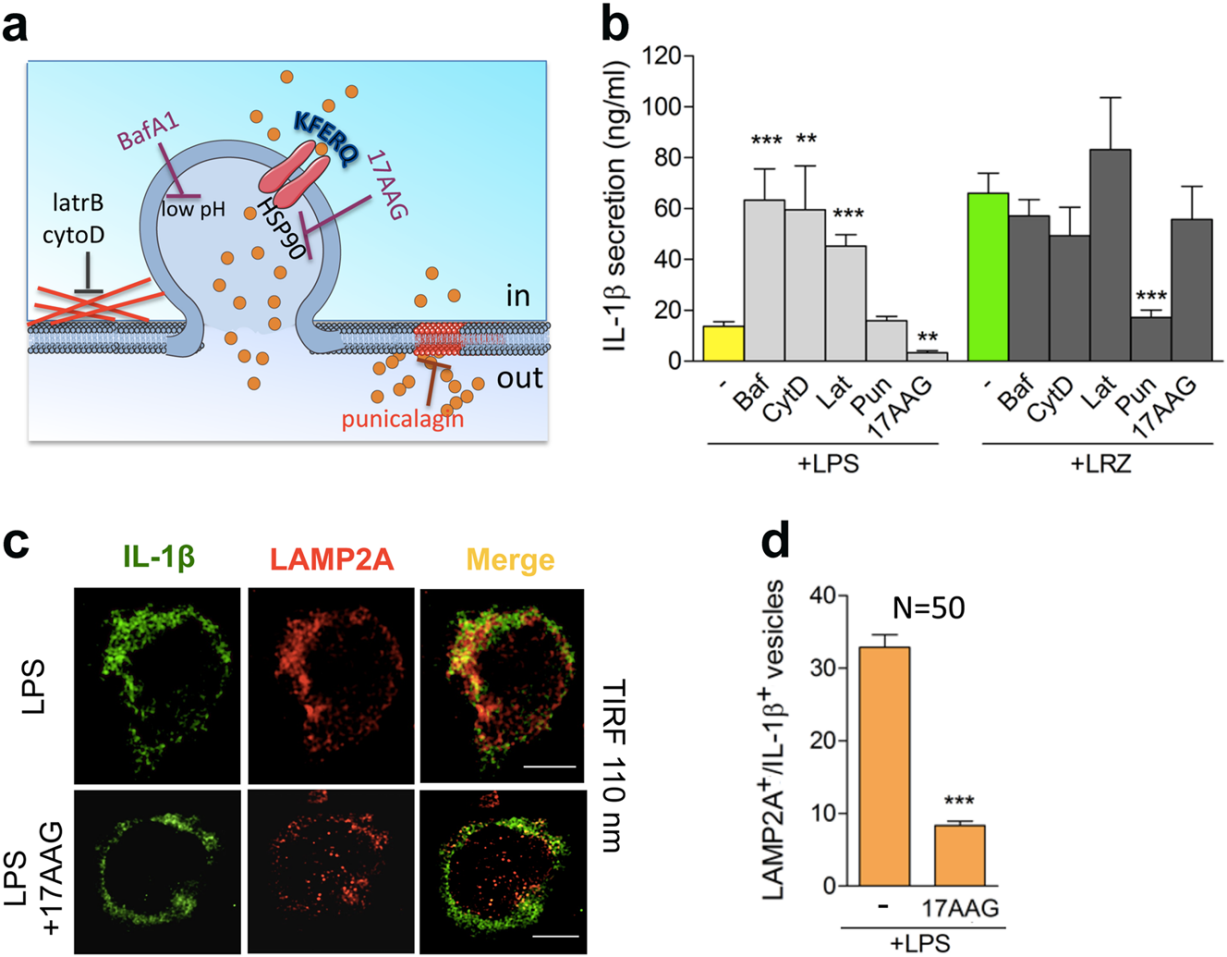
IL-1β secretion by LPS and LRZ-stimulated monocytes is differently modulated by drugs interfering with specific cell processes. **a** The cartoon summarizes the targets of the drugs used in these experiments. Bafilomycin (BafA1) blocks the proton influx into endolysosomes, cytocalasin D (cytoD) and latrunculin B (latrB) inhibit actin polymerization. All these drugs induce secretory lysosomes exocytosis^8,39,40^. 17AAG is an HSP90 inhibitor reported to prevent IL-1β translocation and secretion^17^. Punicalagin stabilizes lipids in the plasma membrane and inhibit IL-1β secretion in mouse macrophages^41^. **b** IL-1β secreted by LPS and LRZ-stimulated monocytes, untreated or treated with BafA1 (BAF), cytocalasin D (CytoD), latrunculin B (Lat), punicalagin (Pun), 17AAG, quantified by ELISA. *N* = 6. **c, d** LPS or LPS + 17AAG-treated monocytes were co-stained with anti LAMP2A and anti IL-1β Ab and analyzed by TIRF microscopy (penetration depth 110 nm) after 6 h of incubation (*N* = 3). (**c**) Representative LPS and LPS + 17AAG-treated monocytes are shown. Scale bar, 5 μm. **d** TIRF quantification of the number of LAMP2A^+^/IL-1β^+^ vesicles found in monocytes stimulated with LPS or LPS + 17AAG, obtained as in Fig. 4b (*N* = 3). Data information: In **b, d** Data are expressed as ng/ml, mean ± SEM. Unpaired *t*-test was used, and the significance compared with untreated cells is indicated (**P < 0.01, ***P < 0.001)

Genetic manipulation of short-lived and trigger-easy monocytes is difficult and may result in improper effects. Hence, to gain further information on the IL-1β secretory route utilized by LPS or LRZ treated monocytes we used drugs that target different pathways (Fig. 5a). Exocytosis of secretory lysosomes is induced by increasing their endolumenal pH^39^ or reducing the cortical actin density^40^. In agreement, bafilomycin A1, which blocks proton influx into endolysosomes, and cytocalasin D and latrunculin B, which inhibit actin polymerization, strongly induced IL-1β secretion by LPS-activated monocytes but had little if any effect on triple-stimulated cells (Fig. 5b). In agreement with the proposed involvement of HSP90 in IL-1β translocation across the lysosomal membrane^41^, fewer LAMP2A^+^/IL-1β^+^ vesicles were detected in LPS-stimulated monocytes following pharmacological inhibition of HSP90 (17AAG, Fig. 5c, d); consequently, IL-1β secretion was decreased in LPS-stimulated but not in LRZ-stimulated monocytes (Fig. 5b). Finally, punicalagin, a compound that stabilizes lipids on the plasma membrane and prevents ATP-induced IL-1β secretion^40^, strongly inhibited LRZ-driven but not LPS-induced secretion (Fig. 5b). The drugs did not significantly affect accumulation of intracellular pro-IL-1β (Fig. S4), implying post-translational regulation of IL-1β transport.

### Exuberant IL-1β secretion by LPS-treated CAPS monocytes occurs via the GSDMD pathway

**Fig. 6:**
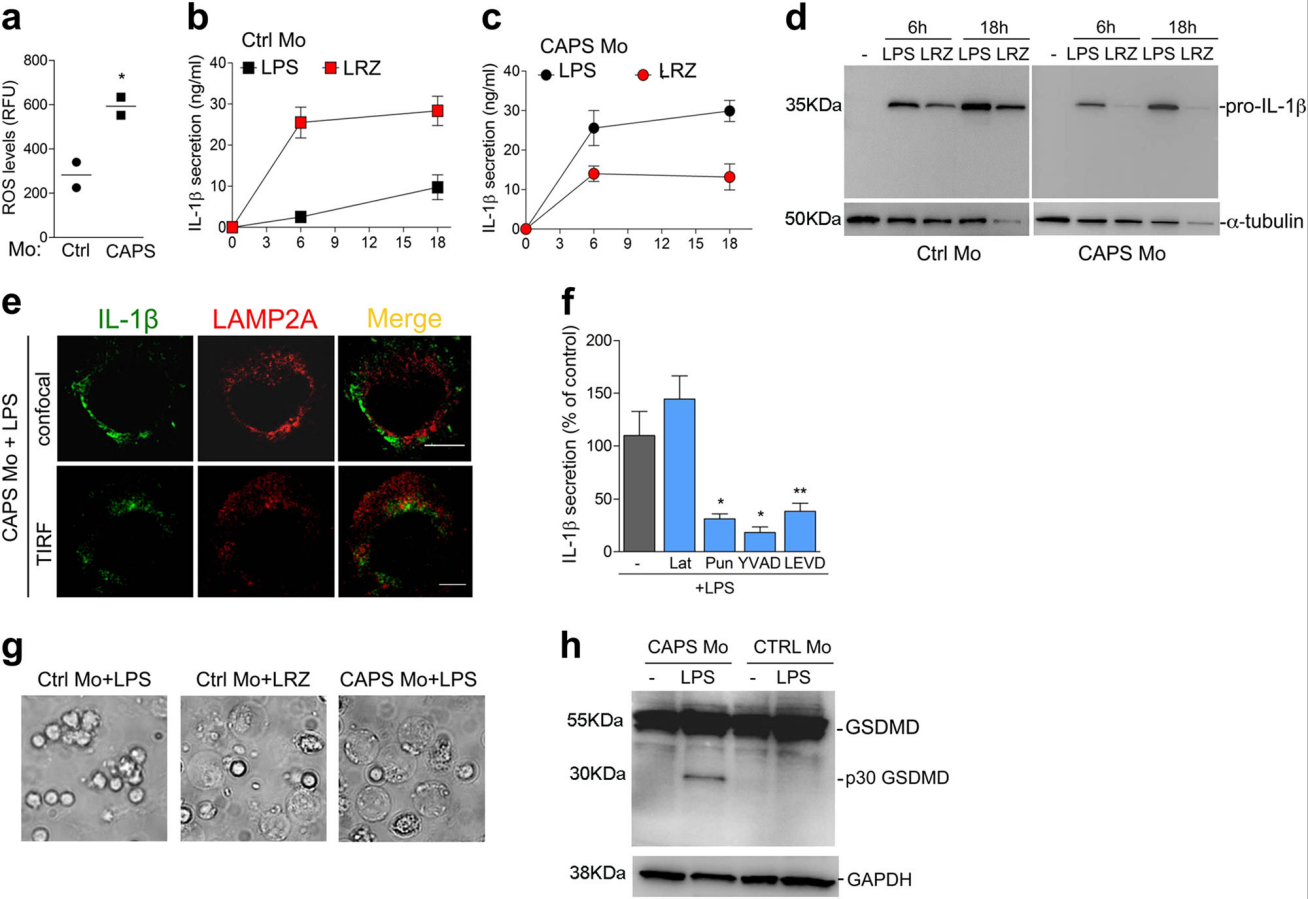
LPS alone is sufficient to trigger GSDMD cleavage and rapid IL-1β secretion in monocytes from CAPS patients **a** Intracellular ROS accumulation was quantified by H_2_DCF-DA staining in unstimulated monocytes (Mo) from two healthy donors (Ctrl Mo) and two CAPS patients. The data are expressed as relative fluorescence units (RFU), and confirmed previous data obtained on a larger number of CAPS and control monocytes^33,34^. **b, c** Kinetics of IL-1β secretion in LPS or LRZ stimulated monocytes from healthy donors (Ctrl Mo) (**b**) or patients (CAPS Mo) (**c**) quantified by ELISA. Average of 5 experiments ± SEM. **d** Accumulation of pro-IL-1β in monocytes from CAPS patients or healthy donors. A representative Western blot out of 3 performed is shown. **e** Confocal [upper panel, one single stack of Z-stack series is shown (Z = 6)] and TIRF (lower panel, penetration depth = 110 nm) microscopy analyses of monocytes from a representative CAPS patient (out of three examined) stimulated for 5 h with LPS. Scale bar, 5μm. **f** IL-1β secreted by LPS-stimulated monocytes from CAPS patients, incubated in the absence or presence of latrunculin B (Lat), punicalagin (Pun), Ac-YVAD or Z-LEVD as indicated, was quantified by ELISA. Data are expressed as percent of IL-1β secretion relative to untreated cells. *N* = 6, mean ± SEM. **g** Live cell microscopy images of monocytes from healthy donors (Ctrl Mo) or CAPS patients (CAPS Mo) exposed for 6 h to LPS or LRZ as indicated. CAPS monocytes stimulated with LRZ died in the first 3 h from stimulations and detached from the well (not shown). *N* = 3 **h** This representative Western Blot (out of 3 performed) compares GSDMD cleavage and total levels in cell monocytes from CAPS patients or healthy donors, after 5 h with or without LPS. Data information: In **a, f** Unpaired *t*-test was performed, and the significance compared with untreated cells is indicated (*P < 0.05, **P < 0.01)

CAPS is a rare autoinflammatory syndrome linked to mutations in the NLRP3 gene^3^. Upon stimulation with low amounts of a single TLR agonist, monocytes from CAPS patients secrete high levels of IL-1β ^33^ responsible for the devastating inflammatory manifestations that hallmark this condition^3^. We then studied peripheral blood monocytes from five CAPS patients bearing mutant NLRP3 protein. The various NLRP3 mutations, as well as demographic features, onset of disease and ongoing therapeutic treatment of patients are shown in Table 1. The levels of ROS detected in monocytes of two CAPS patients were much higher than in healthy controls (Fig. 6a), confirming our previous findings on a larger cohort of CAPS patients^33,34,42^ and indicating that CAPS monocytes are under stress at baseline, in the absence of any stimulation. After LPS stimulation, CAPS monocytes display a strong and accelerated secretion of IL-1β, similar to that observed in LRZ-stimulated monocytes from healthy donors (Fig. 6b, c). Like in hyperstimulated healthy monocytes, this feature is most likely due to the high ROS levels, as preventing ROS biogenesis by DPI inhibited IL-1β secretion by both LPS-stimulated CAPS monocytes^33^ and LRZ-stimulated healthy monocytes (31 and see Fig.1e). LRZ stimulation of CAPS monocytes inhibited pro-IL-1β accumulation, processing and secretion (Fig. 6c, d) possibly due to early induction of oxidative stress by hyperstimulation^34,41^.

**Table 1:** Demographic features and ongoing treatment of CAPS patients

Patients	Sex	*NLRP3* mutation	Disease onset	Age (years)	Treatment
1	M	T348M	6 months	30,2	Canakinumab 300 mg every 4 weeks
2	F	E525K	15 months	24	Canakinumab 150 mg every 8 weeks
3	M	M406I	Birth	15,75	Canakinumab 4 mg/kg every 4 weeks
4	F	E567K	Birth	6,9	Anakinra 7 mg/kg/day
5	M	D303N	2 weeks	2,9	Anakinra 2 mg/kg/day

We then investigated whether in LPS-stimulated CAPS monocytes IL-1β follows the vesicular or the GSDMD-mediated pathway. As shown in Fig. 6e, confocal (upper panel) and TIRF (lower panel) analyses revealed minimal co-localization of LAMP2A and IL-1β in LPS-triggered monocytes from CAPS patients, like in LRZ-stimulated healthy monocytes. Drugs modulating lysosomal-mediated secretion, such as latrunculin B, only slightly affected IL-1β secretion by LPS stimulated CAPS monocytes (Fig. 6f) whereas punicalagin strongly impaired IL-1β release (Fig. 6f) like in hyperstimulated healthy monocytes (see Fig. 5b). In addition, single stimulation with LPS, unable to induce pyroptosis in healthy monocytes, clearly caused pyroptotic cell death in CAPS monocytes (Fig. 6g). Consistently, the cleaved p30 form of GSDMD was clearly induced by LPS alone (Fig. 6h) and Z-LEVD decreased IL-1β secretion by LPS-activated monocytes from CAPS patients (Fig. 6f), even more efficiently that in LRZ-stimulated monocytes from healthy donors (see Fig. 1h, j). Thus, the extracellular TLR stimulation by LPS alone is sufficient to trigger GSDMD-dependent IL-1β secretion in CAPS monocytes.

## Discussion

The main finding of study obeys to the fundamental tenet that the intensity of inflammatory responses should be proportional to their causes. Thus, monocytes use different mechanisms to release IL-1β depending on the strength of the proinflammatory stimuli they perceive. Triggering TLR4 alone induces slow secretion of IL-1β^30–32^ via exocytosis of secretory lysosomes, followed by apoptosis. Multiple TLR triggering induces cleavage of GSDMD with fast and intense IL-1β secretion and leads to pyroptosis. The two mechanisms correlate with different redox responses: multiple TLR stimulation activates stronger production of ROS than LPS alone^32^. Inhibition of ROS production prevents both GSDMD cleavage and IL-1β secretion, suggesting a causal role of the insurgent oxidative stress in the generation of the p30 porogenic fragment. Accordingly, addition of a pro-oxidant drug such as As_2_O_3_ to LPS led to GSDMD-mediated secretion.

Primary human monocytes represent the prototypic model to study IL-1β secretion, as they are the real professionals in this activity. The individual variability in the rate of IL-1β secretion^43^ is well compensated by the high proximity to the physiologic process. Indeed, the use of primary monocytes avoids artifacts often observed in cultured cell lines whose strong antioxidant systems alter IL-1β synthesis, processing and secretion^25^. Plasmid and siRNA transfection is challenging in primary monocytes. However, considering that transfection may induce severe cell stress and hence activate inflammasomes and IL-1β secretion^44–47^, the use of non genetically-modified primary cells reduces the risk of erroneous interpretations and yields precise information on the physiologic mechanisms governing the secretion of this cytokine.

Several lines of evidence indicate the existence of two non-mutually exclusive mechanisms of IL-1β secretion in primary monocytes. First, cleaved GSDMD is present only in LRZ-stimulated monocytes; in contrast, the number of LAMP2A^+^/IL-1β^+^ vesicles is significantly higher in LPS-stimulated than in LRZ-stimulated monocytes from all the healthy donors examined. The concentration beneath the plasma membrane suggests that LAMP2A^+^/IL-1β^+^ lysosomes are undergoing exocytosis. Second, while a fraction of LPS-stimulated monocytes undergo apoptosis, most LRZ-stimulated cells die by pyroptosis. In hyperstimulated (but not in singly-stimulated) monocytes, PI positivity was high at 6 h, when IL-1β secretion is strong, indicating that GSDMD pore formation occurs in concomitance with IL-1β secretion. At this time point, LDH release by hyperstimulated monocytes was low and similar to LPS-stimulated monocytes, even though they secreted 10 times more IL-1β. This implies that pores are initially highly selective. Only later, when IL-1β secretion is not anymore increasing, more LDH is highly released by triply-triggered monocytes. Differently, LDH release by LPS-activated monocytes remains low, in agreement with their apoptotic cell death. Thus, neither overt pyroptosis nor apoptosis seems required for IL-1β secretion^24,36^. Third, drugs inducing secretory lysosome exocytosis increase LPS-induced but not LRZ-induced secretion. Of note, blocking HSP90, which assists the lysosomal translocation of cytosolic proteins during CMA^38^, reduced both IL-1β secretion and vesicular content in single stimulated cells, suggesting the involvement of CMA in LPS-induced IL-1β secretion^17^. Finally, punicalagin^41^ inhibited IL-1β secretion by LRZ-stimulated monocytes only. As the effects of punicalagin are similar to those of knocking out GSDMD^41^, this result supports that LRZ-induced IL-1β secretion is mediated by GSDMD pores.

Generation of the pore-forming domain of GSDMD is attributed to cleavage by caspase 1 or caspase 4/5, although the underlying molecular mechanism is unclear^19,22^. The involvement of caspase 4/5 is confirmed in hyperstimulated monocytes, as caspase-4/5 inhibitors decrease GSDMD cleavage and reduces IL-1β secretion. Caspase 1 is likely to participate, as the simultaneous inhibition of caspases 4/5 and caspase 1 induces a stronger decrease of cleaved GSDMD. However, since inhibition of caspase 1 abrogates pro-IL-1β processing and hence mature IL-1β secretion, it is difficult to estimate to which extent is the decrease of IL-1β secretion due to inhibition of processing and of GSDMD cleavage. Still, secretion of mature IL-1β occurs -and is abrogated by Ac-YVAD- both in single and triple stimulated monocytes, whereas GSDMD cleavage occurs in triple stimulated cells only: thus, at least in LPS stimulated monocytes, caspase 1 is able to process pro-IL-1β, but not to cleave GSDMD. IL-18, like IL-1β, lacks a signal sequence and requires processing by the canonical inflammasome. Notably, the inhibition of caspase 4/5 strongly prevents IL-18 secretion in triply, but not singly-stimulated monocytes, indicating that, under hyperstimulation, GSDMD also mediates the secretion of this cytokine. Conversely, secretion of TNF-α-a cytokine secreted through the classical exocytic pathway - by singly or triply stimulated monocytes is unchanged by the exposure to caspase 1 or caspase 4/5 inhibitors ruling out that these compounds affect other cellular functions.

How caspases 4/5 are activated in this system remains unclear. Direct activation by LPS in the cytosol, as proposed in other cell types^22^ is unlikely because we used extracellular LPS at doses that activate NLRP3 inflammasome generating active caspase 1 and secretion of mature IL-1β^43^ but do not induce GSDMD cleavage in single stimulated monocytes.

Our data indicate that in primary monocytes apoptosis is favored in response to a mild stimulus whereas pyroptosis is the fate after a stressful one. Switch from the apoptotic to the pyroptotic death however occurs if other TLR agonists are added after LPS, in agreement with the observation of a precarious equilibrium between apoptosis and pyroptosis in monocytes and macrophages, due to differential cleavage of GSDMD by inflammatory and apoptotic caspases^48^. The prevalence of apoptosis or pyroptosis in monocytes has relevant consequences, as the latter allows DAMPs release, thus playing as an amplifier of inflammation^37^.

Finally, our study sheds new light into the pathophysiology of CAPS. Single stimulation, which induces vesicular IL-1β secretion in healthy monocytes, triggers the GSDMD-mediated pathway of secretion in monocytes from CAPS patients. Activation of pore-mediated secretion by a single TLR agonist may be due to the basal stress conditions that hallmark CAPS monocytes, which do not express IL-1β but display high level of ROS before TLR stimulation. Stimulation with low amounts of LPS causes a further increase of ROS^33,34^ as it occurs in healthy monocytes only upon hyperstimulation^32^. Our data indicate that the strong ROS production not only activates the classical inflammasome as proposed in different systems^49,50^ but also induces GSDMD cleavage possibly through the activation of non-canonical inflammasomes,. The activation of the pyroptotic pathway may explain the devastating inflammatory manifestations displayed by CAPS patients even in the absence of a detectable noxia. Consistent with our data, a recent study identified IL-1β-producing monocytes susceptible to pyronecrotic cell death, similar to pyroptosis, in patients with Neonatal-Onset Multisystem Inflammatory Disease, the most severe form of CAPS^51^. In addition, Kanneganti et al. recently showed that GSDMD is critical for autoinflammatory pathology in a mouse model of Familial Mediterranean Fever^52^.

**Fig. 7:**
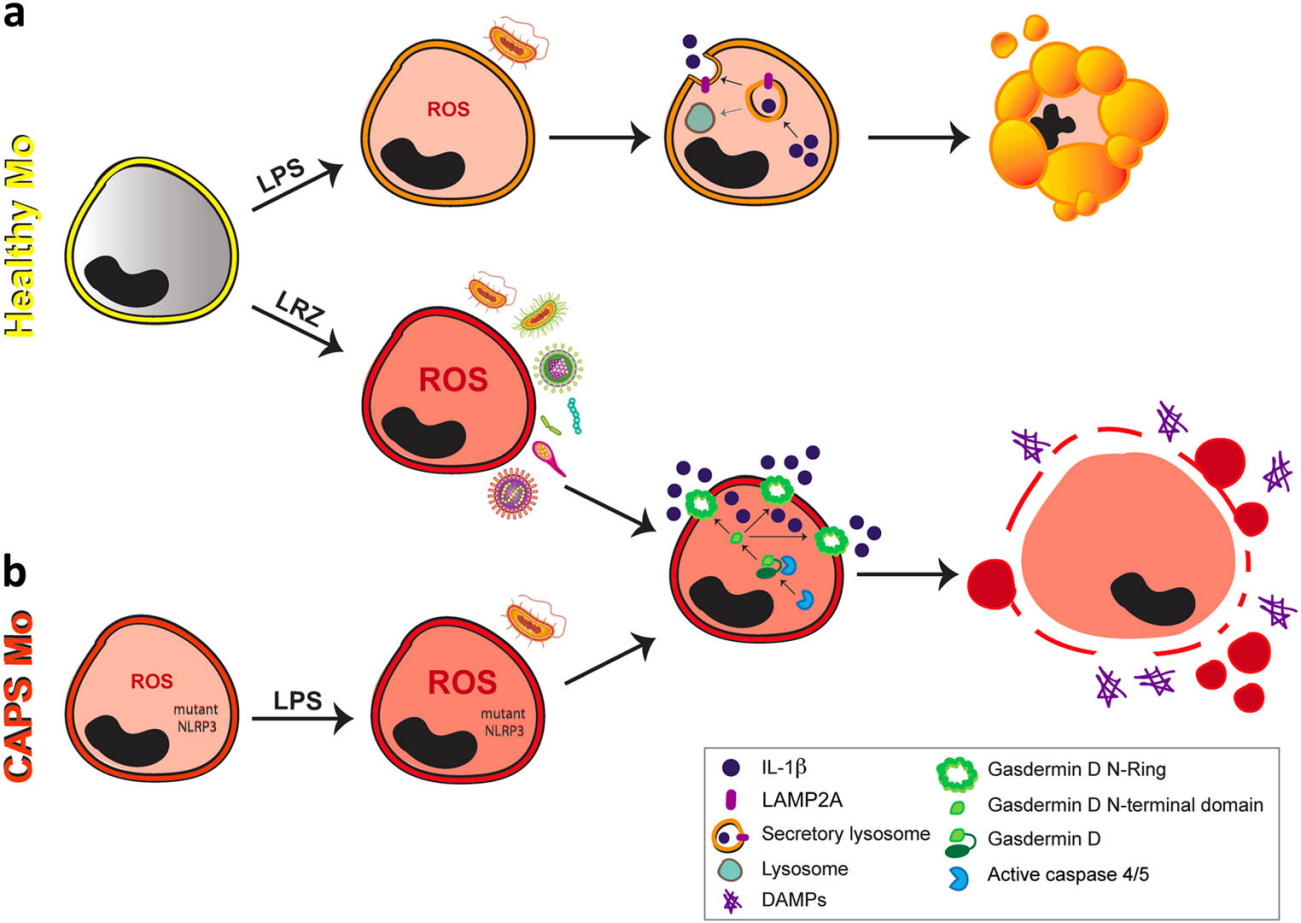
Routes of IL-1β secretion in human monocytes Two different mechanisms for IL-1β secretion can be activated in primary human monocytes depending on the strength of the inflammatory stimulus. **a** In monocytes from healthy donors (**Healthy Mo**), small trauma or low pathogen load (**LPS**) activates a pathway involving secretory lysosomes that allows slow release of IL-1β, followed by apoptotic cell death that switches off the inflammatory response. Differently, a stronger stimulus (**LRZ**) results in **gasdermin D** cleavage with generation of the N-terminal domain that assembles in N-rings with formation of pores through which IL-1β can be externalized: this pathway of secretion is followed by pyroptosis, with membrane ruptures through which DAMPs can leave cells, further amplifying the inflammatory response. **b** Monocytes from CAPS patients (**CAPS Mo**) contain higher level of ROS at baseline that healthy monocytes. This condition enables the activation of the GSDMD-mediated IL-1β secretory pathway and the consequent hypersecretion of IL-1β even after small trauma or low pathogen load (**LPS**). Thus, hyperstimulated healthy monocytes and mildly stimulated CAPS monocytes use the same GSDMD-mediated pathway of IL-1β secretion

The scenario emerging from our study is that the route of IL-1β secretion depends on the overall level of stress induced in monocytes by inflammatory stimuli (Fig. 7). Thus, the secretory lysosome-mediated mechanism prevails in conditions of low pathogen load or small trauma, inducing a self-limiting redox response and allowing a controlled release of IL-1β to restore the homeostasis. Along this line, lysosome exocytosis represents a general strategy used by cells to restore plasma membrane integrity^53^ and prevent the release of DAMP that would amplify inflammation. Accordingly, mildly stimulated monocytes die by apoptosis, a non-inflammatory process (Fig. 7a). However, if noxia persist and increase, like in the case of severe trauma or infection, then pyroptosis-mediated secretion is activated (Fig. 7a). Although this strategy may initially benefits the host, it soon starts a vicious circle of pyroptotic release of IL-1β and additional DAMPs that may lead to death, as it occurs in sepsis or toxic shock. Not only exogenously generated cell stress causes GSDMD cleavage and hypersecretion, but also endogenous cell stress due to genetic alterations, as we showed for CAPS (Fig. 7b), where even little amounts of a single TLR agonist trigger dramatic levels of secreted IL-1β^34^. Thus, GSDMD inhibition may represent a novel potential anti-inflammatory strategy in acquired and inherited severe inflammatory conditions.

## Methods and materials

### Chemicals and TLR agonists

LPS (from *Escherichia coli* 0111:B4), zymosan, dibenziodolium chloride (DPI), As_2_O_3_, bafilomycin A1, latrunculin B, cytocalasin D, and punicalagin were from Sigma-Aldrich; R848 was from Invivogen; 2′,7′-dichlorofluorescein diacetate (H_2_DCF-DA) was from Invitrogen; 17AAG and Ac-YVAD-CMK were from Cayman Chemical and Z-LEVD-FMK was from BioVision; CD14 MicroBeads was from Miltenyi Biotec.

### Cell preparation and culture

Peripheral blood mononuclear cells were isolated from freshly drawn heparinized blood by Ficoll-Paque (Sigma-Aldrich) gradients and plated at 4 × 10^6^ per well in 24 well plates. After 30 min incubation, 4 × 10^5^ monocytes were found adherent to the plastic well. Adherent cells were activated with LPS (100 ng/ml) alone or in combination with R848 (5 μg/ml) and zymosan (20 μg/ml) (LRZ) at 37 °C in RPMI 1640 medium supplemented with 5% FCS (Euroclone)^32^. When indicated, the following substances were added to cultures: bafilomycinA1 (1 μM) latrunculin B (1 μM), cytocalasin D (1 μM), 17AAG (400 nM), punicalagin (20 μM), Ac-YVAD-CMK (20 μM), Z-LEVD (2 μM), DPI (20 μM), As_2_O_3_ (0,5 and 1 μM). At the end of the culture periods, supernatants were recovered and cells were lysed for Western blot as described^9,34^.

### Patients and blood samples

Five CAPS patients positive for mutations of the NLPR3 gene and five age-matched healthy controls were studied in parallel. Demographic features, NLRP3 mutation, duration of the disease and ongoing treatment of the five CAPS patients are shown in Table 1. Blood samples were taken after informed consent by patients or parents, approved by the ‘G Gaslini’ Ethical Board, and monocytes were stimulated as described^34^.

### Western blot analyses

Triton X-100 cell lysates and trichloroacetic acid–concentrated supernatants from monocytes were resolved by 12% SDS/PAGE and electrotransferred. Filters were probed with 3ZD anti-IL-1β mAb (IgG1; obtained from the National Cancer Institute Biological Resources Branch), anti-GSDMDC1 (Novus Biologicals), anti-human LAMP2A Ab (Abcam), anti human α-tubulin mAb (Sigma-Aldrich) and anti-GAPDH (Novus Biologicals), followed by the relevant secondary Ab (Dako) and developed with ECL-plus (BioRad) (GE Healthcare), as described^9,34^. The total amounts of cell culture supernatants and 10 μg of proteins of cell lysates (corresponding to about 25% of the total cell lysates) were loaded for IL-1β experiments. For GSDMD and LAMP2A detections 40 μg of cell lysate were loaded.

### ELISA analyses

IL-1β, IL-18, and TNF-α in supernatants recovered at different time points was determined using the ELISA kit from R&D Systems for human IL-1β and TNF-α and from MBL for human IL-18.

### Intracellular ROS detection

Monocytes (4 × 10^5^/well) unstimulated or stimulated with the different PAMPs for 1 h were incubated 30 min at 37 °C with 10 μM H_2_DCF-DA. Cells were then washed and lysed in 50 μL of 0.2% Triton X-100 in PBS. Fluorescence was measured in cell lysates in a microplate fluorometer (excitation 480 nm; emission 530 nm) as described previously^32,34^. The fluorescence signal intensity was normalized vs. the protein content in each sample, evaluated with the Bio-Rad protein assay kit.

### Live cell imaging

Peripheral blood mononuclear cells were plated at 5 × 10^6^/well in 24 mm glass coverslips (Marienfeld, Germany) for 30 min, washed in PBS and incubated in complete medium at 37 °C with different stimuli. Time-lapse photographs were then taken every 3 min for up to 6 h using a Zeiss Axiovert S100 TV2 microscope (Zeiss) equipped with a 63 × /1.4 lens and a Hamamatsu OrcaII-ER camera (Hamamatsu City). Images were analyzed using ImageJ or Oko-vision (Okolab) softwares^54^. Monocytes stimulated for 6 h with LPS or LRZ or for 3 h with LPS and then with R848 and zymosan for 3 h were stained for 10 min with PI (5μg/ml) and images were recorded with the software LASX on a Leica DMI6000 microscope (Leica Microsystems, Mannheim, Germany) with a 63 × /1.45 objective and a sCMOS camera (Hamamatsu Flash ORCA 4.0).

### Immunofluorescence analyses

Monocytes, plated on 24 mm glass coverslips (Marienfeld, Germany), were incubated at 37 °C in the presence of LPS or LRZ for 6 h (*N* = 6). Samples were then washed with PBS, fixed and permeabilized with 4% paraformaldehyde and 0.5% TritonX-100 in PBS. After washing and saturation with PBS supplemented with 2% (w/v) BSA (Sigma), cells were labeled with primary antibodies against IL-1β (mAb 3ZD) and LAMP2A (rabbit, Abcam, UK) in PBS/BSA, followed by incubation with specific secondary antibodies labeled with Alexa-546 (Life Technologies, USA) or Alexa-647 (Invitrogen, USA). Images were obtained using a Leica TCS SP8 scanning confocal microscope equipped with a 63 × /1.4 oil objective and LAS AF software with a Z-stack of 170 nm. Reconstructed 3D volume was obtained using ImageJ. For TIRF analyses, cells were examined under a Leica SR Ground State Depletion (GSD), microscope, with a 160 × /1.45 objective and an sCMOS camera (Hamamatsu Flash ORCA 4.0). In order to enhance the signal vs. background and identify/quantify the vesicles, images were filtered with a Laplacian of Gaussian filter (using the FeatureJ-Laplacian plugin of ImageJ with a smoothing scale parameter of 1), before applying a histogram based threshold.

### Super-resolution microscopy

Super-resolution localization imaging of fixed and double-immunostained cells was obtained by direct stochastic optical reconstruction microscopy (dSTORM^55^), using a Ground State Depletion (GSD) microscope (Leica SR GSD, Leica Microsystems). The microscope is equipped with two solid state lasers of 532 nm and 642 nm and an oil immersion objective lens (HCX PL APO 160 × /1.45). The images were collected with LAS X Software (Leica) on an Andor iXon3 897 EMCCD camera, with a final optical pixel size of 100 nm. The lateral drift was minimized by the Suppressed Motion (SuMo) stage. The two color-measurements of immunostained LAMP2A and IL-1β were carried out sequentially in a buffer of 10 mM mercaptoethylamine (Sigma Aldrich) in PBS (pH 8.0) including 10% (w/v) glucose, 0.4 mg/mL glucose oxidase (Sigma-Aldrich) and as oxygen scavenger 55 μg/mL catalase (Sigma-Aldrich). LAMP2A was recorded first using the 642 nm laser for depletion and acquisition; subsequently the 532 nm laser was used for depletion and acquisition of IL-1β. To limit the localization of LAMP2A, we used TIRF for acquisition setting, which allows imaging within a defined depth (250 nm) from the plasma membrane. The list of the detected molecules in the two channels was used to compute the cross-correlation (C) of the two images with MatLab as described:^56^ the cross correlation function reports the increased probability of finding a second localized signal at a certain distance away from a given localized signal. When a signal is randomly distributed, the cross-correlation function describing its organization is ~1. Ten representative Regions Of Interest (ROIs) (2 × 2 μm^2^) were chosen in the peripheral region of 6 cells (for each treatment) in 4 independent experiment (*N* = 4). All the results were pooled together with a pair statistical test. For visualization purposes, the super-resolution images were reconstructed by rendering each of the detected molecules as a Gaussian distribution, with width equal to the localization precision of each event.

### Cytofluorimetry

Monocytes were isolated from PBMCs using CD14 MicroBeads (Miltenyi Biotec) and activated with LPS or LRZ. Cytofluorimetry was performed as indicated^57^. Cells were stained with anti-human LAMP2A Ab (Abcam) followed by the anti-rabbit FITC secondary Ab (Bethyl Laboratories, Inc). Samples were acquired using a CyAn flow cytometer (Beckman Coulter) and analyzed by the Summit V4.3 software (DAKO)^32^.

### LDH release

The release of LDH was measured by the colorimetric Cytotox 96 Non-Radio Cytotoxicity Assay from Promega.

### Statistical analyses

Data are presented as mean ± SEM from the number of assays indicated. Data were analyzed by an unpaired two-tailed Student’s t-test or a pair t-test to determine the difference between two groups, or by one-way ANOVA followed by Bonferroni post test to determine the differences among more than two groups, using GraphPad Prism software. Significance is expressed as *P < 0.05, **P < 0.01, ***P < 0.001.

## Electronic supplementary material

Fig. S1

Fig. S2

Fig. S3

Fig. S4

Movie S1

Movie S2

Movie S3

Movie S4

Movie S5

Movie S6

supplementary figure legends
