# Supplementary figures and images for: Progressive waves of IL-1β release by primary human monocytes via sequential activation of vesicular and gasdermin D-mediated secretory pathways
Source: Cell Death Dis. 2018 Oct 23;9(11):1088. doi: 10.1038/s41419-018-1121-9 (PMC6199333; doi:10.1038/s41419-018-1121-9)

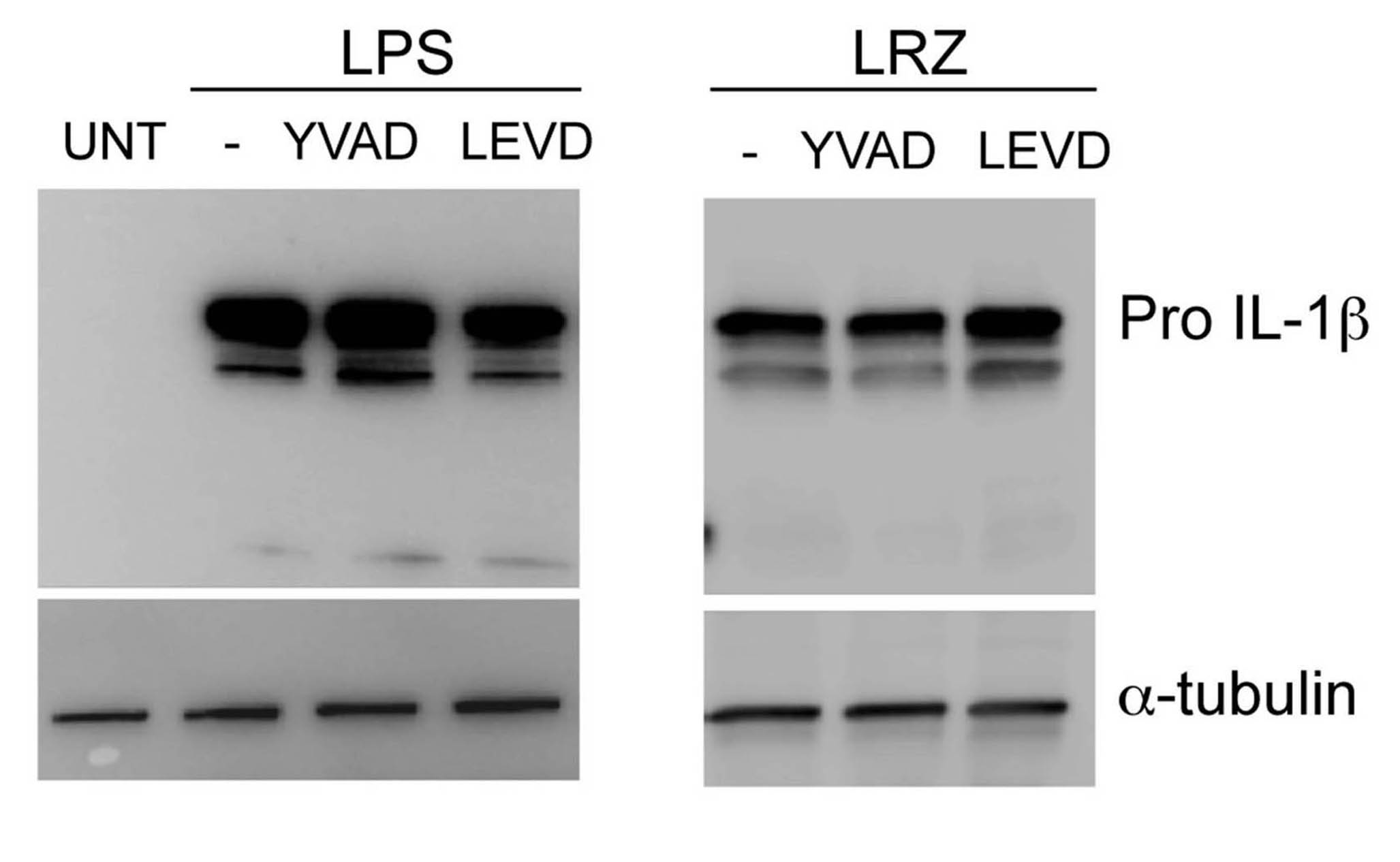

Supplement: Supplementary file 1 — Fig. S1 [file 41419_2018_1121_MOESM1_ESM.jpg]

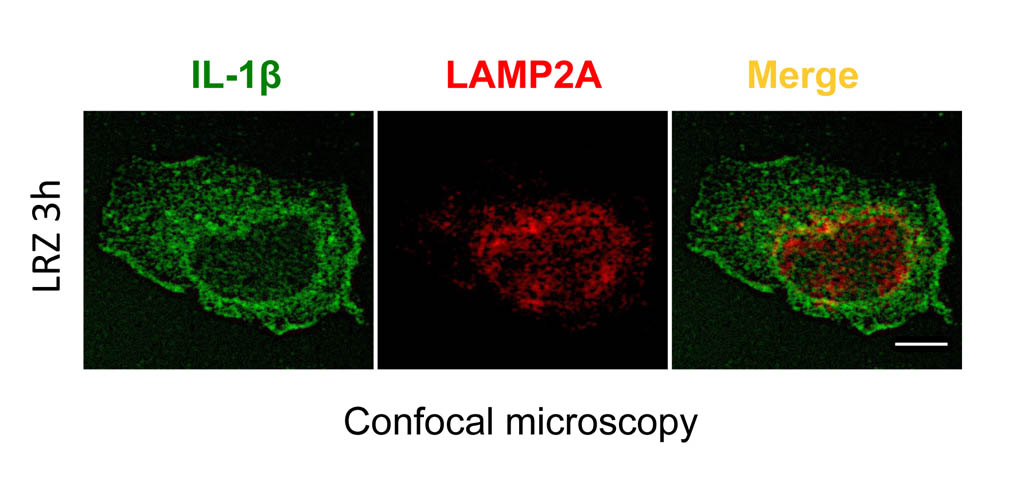

Supplement: Supplementary file 2 — Fig. S2 [file 41419_2018_1121_MOESM2_ESM.jpg]

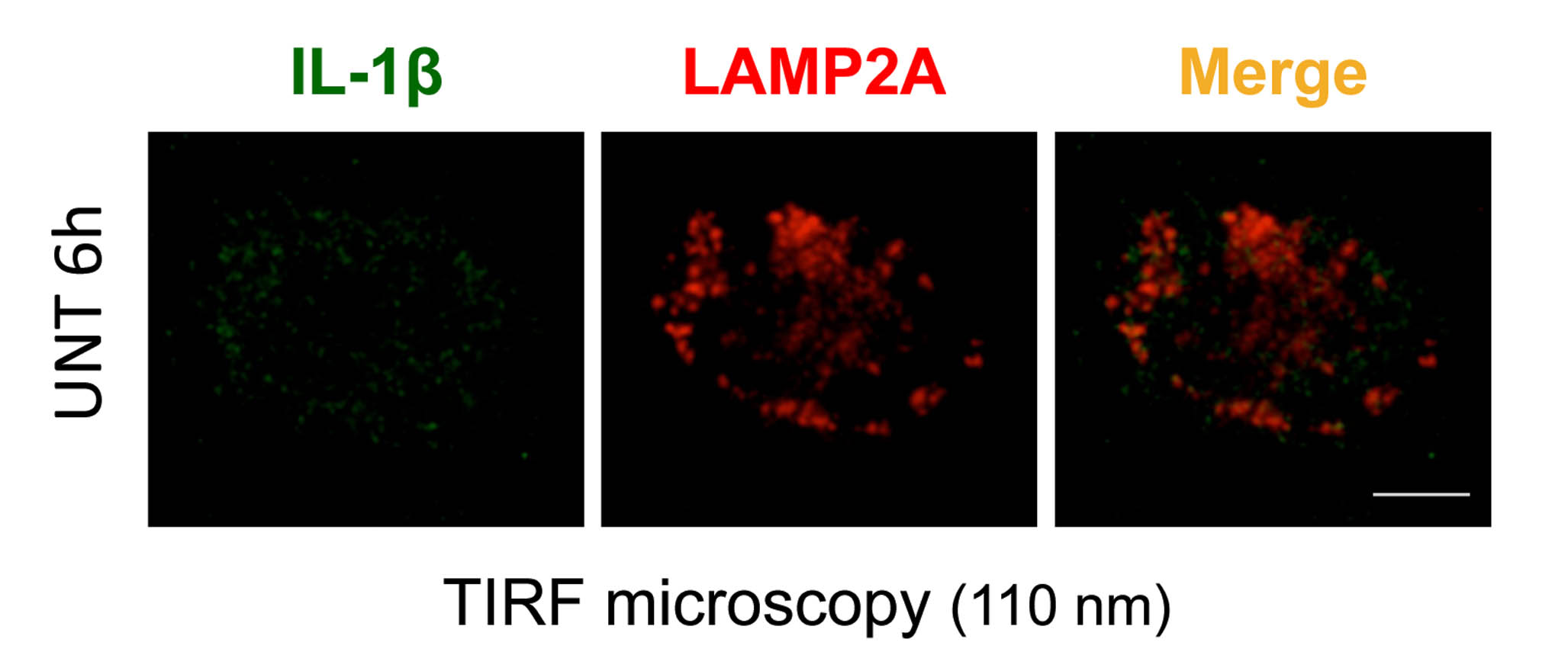

Supplement: Supplementary file 3 — Fig. S3 [file 41419_2018_1121_MOESM3_ESM.jpg]

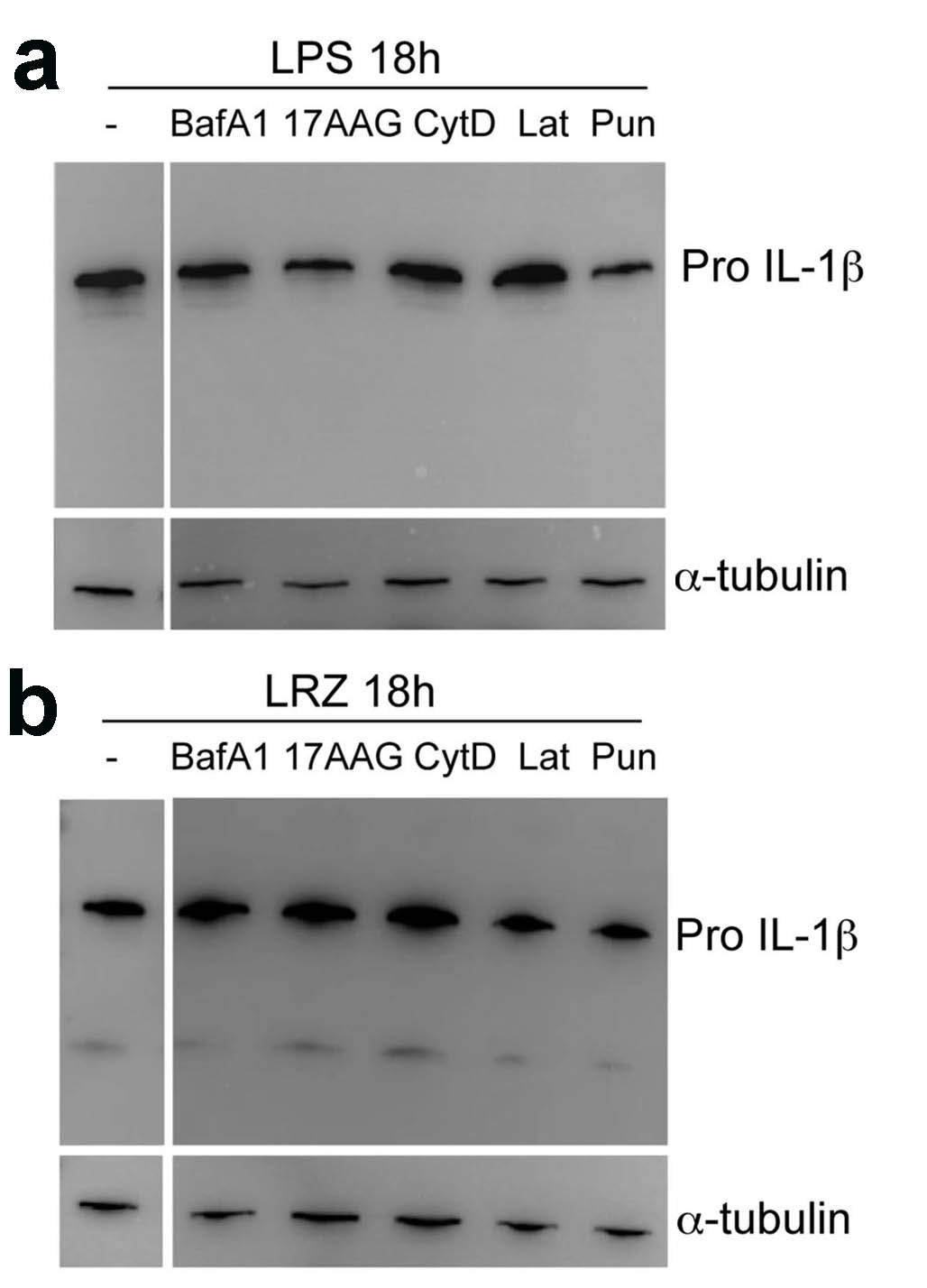

Supplement: Supplementary file 4 — Fig. S4 [file 41419_2018_1121_MOESM4_ESM.jpg]
