# Supplementary material for: Progressive waves of IL-1β release by primary human monocytes via sequential activation of vesicular and gasdermin D-mediated secretory pathways
Source: Cell Death Dis. 2018 Oct 23;9(11):1088. doi: 10.1038/s41419-018-1121-9 (PMC6199333; doi:10.1038/s41419-018-1121-9)
Supplement: Supplementary file 11 — supplementary figure legends [file 41419_2018_1121_MOESM11_ESM.docx]

**Supplementary Figure Legends**

**Supplementary Figure 1.** **Caspase inhibitors do not affect pro-IL-1β intracellular content**

Pro-IL-1β in cell lysates from monocytes unstimulated (Unt) or stimulated with LPS (left panel) or LRZ (right panel) and treated with or without YVAD or zLEVD, as indicated. α-tubulin is shown as a loading control. A representative Western blot is shown out of 3 performed.

**Supplementary Figure 2. LRZ-stimulated monocytes contain LAMP2A+ lysosomes not co-localizing with IL-1β**

Confocal microscopy of a representative monocyte stimulated 3 h with LRZ, stained for IL-1β and LAMP2A as indicated (N=3). Scale bar, 5μm. One single stack of Z-stack series is shown (Z=7).

**Supplementary Figure 3. Unstimulated monocytes do not express IL-1β but display LAMP2A^+^ lysosomes**

TIRF microscopy of a representative monocyte unstimulated for 6h and stained for IL-1β and LAMP2A as indicated (N=3). As expected, primary human monocytes do not produce IL-1ß unless they are stimulated via TLR agonists (47). The distribution of LAMP2A containing vesicles is similar to LRZ-treated monocytes.

**Supplementary Figure 4.** **Drugs affecting IL-1β secretion do not influence pro-IL-1β intracellular content**

Pro-IL-1β in cell lysates from monocytes stimulated with LPS (**a**) or LRZ (**b**) and treated with different drugs, as indicated. α-tubulin is shown as a loading control. A representative Western blot is shown out of 3 performed.

**Supplementary Movie Legends**

**Supplementary Movie 1**. Time-lapse microscopy of monocytes stimulated with LRZ for 6 h (one representative experiment out of 3 performed). Frames were collected every 3 min. Period time from 3 h to 5 h from the beginning of the incubation is shown. Scale bar, 5μm.

**Supplementary Movie 2**. Time-lapse microscopy of monocytes stimulated with LPS for 6 h (one representative experiment out of three performed). Frames were collected every 3 min. Period time from 4 h to 6 h from the beginning of the incubation is shown. Scale bar, 5μm

**Supplementary Movie 3**. Time-lapse microscopy of untreated monocytes cultured for 6 h (one representative experiment out of 3 performed). Frames were collected every 3 min. Period time from 4 h to 6 h from the beginning of the incubation is shown. Scale bar, 5μm.

**Supplementary Movie 4.**Time-lapse microscopy of monocytes stimulated 3h with LPS followed by 3 h with R848 and zymosan (one representative experiment out of 3 performed). Frames were collected every 3 min. Period time from 4 h to 6 h from the beginning of the incubation is shown. Scale bar, 5μm.

**Supplementary Movie 5**. Confocal analysis tridimensional reconstruction of a Z-series (ImageJ) of a representative image of a monocyte stimulated for 6 h with LPS and decorated with anti-IL-1β and anti-LAMP2A Ab. Scale bar, 5 μm.

**Supplementary Movie 6**. Confocal analysis tridimensional reconstruction of a Z-series (ImageJ) of a representative image of a monocyte stimulated for 6 h with LRZ and decorated with anti-IL-1β and anti-LAMP2A Ab. Scale bar, 5 μm.
